# Sexual Dimorphism in Preterm Milk: A Step Toward the Individualized Fortification of Mother’s Own Milk?

**DOI:** 10.3390/nu17162644

**Published:** 2025-08-15

**Authors:** Inês Rodrigues, Luís Proença, Renata Ramalho, Daniel Virella, Luís Pereira-da-Silva, Manuela Cardoso

**Affiliations:** 1 Nutrition Laboratory, Egas Moniz Center for Interdisciplinary Research (CiiEM), Egas Moniz School of Health & Science, 2829-511 Almada, Portugal; ines16.rodrigues@gmail.com (I.R.); rramalho@egasmoniz.edu.pt (R.R.); 2 Egas Moniz Center for Interdisciplinary Research (CiiEM), Egas Moniz School of Health & Science, 2829-511 Almada, Portugal; lproenca@egasmoniz.edu.pt (L.P.); maria.cardoso5@ulssjose.min-saude.pt (M.C.); 3 Neonatology Unit, Maternidade Dr. Alfredo da Costa, Unidade Local de Saúde São José, Centro Clínico Académico de Lisboa—CCAL, 1069-089 Lisbon, Portugal; danielvirella@ulssjose.min-saude.pt; 4 CHRC—Comprehensive Health Research Centre, Nutrition Group, NOVA Medical School, Universidade Nova de Lisboa, 1169-056 Lisbon, Portugal; 5 Medicine of Woman, Childhood and Adolescence Academic Area, NOVA Medical School, Universidade Nova de Lisboa, Centro Clínico Académico de Lisboa—CCAL, 1169-056 Lisbon, Portugal; 6 Nutrition Unit, Maternidade Dr. Alfredo da Costa, Unidade Local de Saúde São José, Centro Clínico Académico de Lisboa—CCAL, 1069-089 Lisbon, Portugal

**Keywords:** human milk composition, preterm milk, sex dimorphism, singleton birth, twin birth

## Abstract

*Introduction:* Several factors can affect the composition of a mother’s milk, including the infant’s sex, gestational age, and single or twin delivery. We aimed to determine the association of the offspring’s sex with the macronutrient and energy content in preterm milk, during the first six weeks postpartum. *Methods*: This is a retrospective, monocentric, cohort study of lactating mothers who delivered before 37 weeks at a referral tertiary maternity. A mid-infrared human milk analyzer was used. *Results:* From 174 mothers, 360 milk samples were obtained. In the milk for singletons, the mature milk for females was significantly richer in total energy, fat, and protein than the milk for males; in advanced lactation, the milk for males was richer in protein than the milk for females. The transitional milk for male twins was significantly richer in fat and energy than the milk for male singletons; mature milk for male twins was richer in energy, carbohydrates, and protein than the milk for singletons. The mature milk for female twins during the fourth week postpartum was significantly richer in fat and total energy than the milk for singletons. *Conclusions:* This study provides information on how the number of delivered fetuses and the infant’s sex affects the composition of preterm milk.

## 1. Introduction

### 1.1. Feeding Preterm Infants with Mother’s Own Milk

Recent advances in the nutritional support of preterm infants have improved their survival rates and health outcomes both in the short [1] and long [2] term.

A mother’s own milk (MOM) is the first option for feeding very preterm infants, due to its short- and long-term benefits [3,4]. However, the macronutrient content of MOM does not meet the high nutritional requirements of growing preterm infants [4]. Therefore, MOM should be supplemented with human milk fortifiers to prevent nutritional deficits and risks of growth restriction and neurocognitive impairment [4].

The most common methods used in clinical practice are standard fortification, adjusted fortification, and target fortification [5]. In the first two methods, the macronutrient and energy content of breast milk is estimated using available literature data. In target fortification, the breast milk is supplemented with nutrients based on measurements of its macronutrient content [5,6,7]. If MOM is unavailable, donor human milk (DHM) is the second choice, as long as a human milk bank (HMB) is available [4].

### 1.2. Sexual Dimorphism in Macronutrient and Energy Content of Breast Milk

Some studies have examined the impact of sexual dimorphism on the macronutrient and energy content of full-term breast milk [8,9,10,11]. However, data on the specific nutritional composition of preterm breast milk according to the sex of the offspring are scarce. A prospective study by Fischer Fumeaux et al. [12] assessed several factors potentially influencing the composition of breast milk. It was found that the milk from mothers of male infants, whether born at term or preterm, had a significantly higher energy and fat content at the fifth and seventh weeks of lactation, as well as a trend for higher protein content.

In another prospective study, Khelouf et al. [13] evaluated how the sex of offspring affects the total fat, cholesterol, carbohydrate, lactose, glucose, total protein, whey protein, casein, and total energy content of preterm and term breast milk in various stages of lactation. It was found that colostrum and mature milk from mothers of males had lower carbohydrate and lactose contents, and their mature milk had a higher fat content. Nevertheless, Galante et al. [14] reported in their narrative review that evidence for a sex-specificity in breast milk composition is limited and conflicting.

### 1.3. Other Factors That May Affect Breast Milk Composition

Systematic reviews and/or meta-analyses have evaluated the impact of several factors on the macronutrient content of breast milk, including maternal and obstetric factors, and factors related to the neonate [15,16,17,18].

In relation to preterm breast milk specifically, the stage of lactation may affect its macronutrient and energy content. A systematic review and meta-analysis of 23 studies analyzing the preterm breast milk composition during the first 12 weeks of lactation suggested a progressive decrease in protein content and a progressive increase in fat content [19].

Another factor that may affect the macronutrient and energy content of preterm breast milk is multiple pregnancy, in contrast with single pregnancy [20,21].

### 1.4. What Needs to Be Explored

Because there is no consensus on how sexual dimorphism affects the macronutrient and energy content of preterm breast milk [14], additional research to evaluate this effect is needed. Moreover, the effect of twinning on the composition of preterm milk deserves to be further evaluated [20,21]. This information is all important for determining the potential advantages of optimizing the nutrition of preterm infants fed MOM or DHM by adapting fortification according to sex [22].

## 2. Objective

This study aimed to identify the differences in macronutrient and energy content between the milk of mothers of male and female preterm infants during the first six weeks postpartum, considering whether single or twin newborns were delivered.

## 3. Methods

### 3.1. Setting and Ethical Issues

The study sample included lactating mothers, who have delivered prematurely at a referral, tertiary care maternity, in Lisbon, whose offspring were admitted into the neonatal unit. This maternity counts with its own HMB, where human milk analyses are performed, both for MOM and DHM.

### 3.2. Ethical and Legal Issues

This study was conducted according to the guidelines of the Declaration of Helsinki and was approved by the Institutional Review Board of the Unidade Local de Saúde São José (protocol code I/26136/2025, approved 25 July 2025). The study was pseudonymized, each participant was assigned a code, and the key was held by a single researcher (M.C.). The key code is to be destroyed 12 months after the results of the study are published.

### 3.3. Study Design and Participants

This monocentric cohort study was based on a consecutive, convenience sample of breast milk from lactating mothers who delivered before 37 weeks of gestation between January 2024 and April 2025. A series of milk samples from participants with single or twin pregnancies were included, whose milk composition was analyzed at least in the first week postpartum. Milk samples from mothers with higher multiple pregnancies were not included.

In this study, the unit of interest was the breast milk. The characteristics of mothers and their offspring were treated as exposure factors.

The study began coinciding with the unit’s new practice of regularly analyzing the macronutrient and energy content of preterm infants’ breast milk on a weekly basis, to guide its possible fortification.

Data on mothers’ and infants’ characteristics were retrieved from the pre-existing institutional HMB files, where data from both for MOM and DHM are systematically and prospectively collected. Mothers whose pregnancies were complicated by intrauterine growth restriction were identified as having small-for-gestational-age infants, which is defined as a birth weight below the third percentile on the Fenton 2013 growth charts [23].

### 3.4. Breast Milk Analysis

As described elsewhere [21], mothers were asked to collect their milk every three hours, whenever possible, either at the hospital or at home, and to record the time (date and hour) of each collection. The milk was stored at −25 °C in the maternity’s HMB. A daily set of sequentially collected frozen milk samples from each mother was thawed at 37 °C and mechanically homogenized for infant feeding. A 3 mL sample from this pool, roughly representing its composition within a day, was collected and again frozen for later analysis. The composition of the mothers’ milk was analyzed weekly by the same observer (M.C.). Before analysis, milk samples were thawed by warming to 40 °C and ultrasonically homogenized.

A mid-infrared human milk analyzer (Miris AB, Uppsala, Sweden) was used as a validated method to measure human milk macronutrient and total energy content [24,25]. The milk composition is expressed in densities: kcal/dL of energy and g/dL of total and true protein, fat, and carbohydrates. This study considered weekly measurements of breast milk composition taken during the first six weeks postpartum. The analysis concluded when the infants began to be breastfed directly, when infant formula was introduced, or when the infants were discharged.

The value of macronutrient and energy content relative to each postpartum week is the mean (SD) of the values of the individual samples analyzed in that week.

The following comparisons of breast milk composition were analyzed:

− In single pregnancies, between the milk from mothers of either male or female infants.
− Between the milk from mothers of either singleton or twin male infants.
− Between the milk from mothers of either singleton or twin female infants.

The milk from mothers of mixed-sex twins was not included in the comparative analysis.

According to the weeks of lactation, breast milk was considered colostrum if produced during the first week postpartum, transitional milk if produced during the second week postpartum, and mature milk if produced from the third week postpartum onwards [26].

### 3.5. Statistical Analysis

Data were analyzed using IBM SPSS Statistics v.29 software, by using descriptive and inferential statistical methodologies. Univariate inferential analyses were carried out to comparatively assess the breast milk macronutrient and energy content (total protein, true protein, carbohydrate, fat, and total energy content) for the different weeks postpartum (from 1st to 6th week), according to the newborn sex and type of pregnancy (singleton/twin). The inferential analyses were based on a parametric comparative statistical test (t-Student), after validating the applied assumptions, namely data adequacy to normality and homoscedasticity. A 5% significance level was set in the inferential analyses.

## 4. Results

Overall, 360 samples of milk were obtained from a total of 174 mothers. Of those milk samples, 241 (67%) came from 115 mothers of singletons, 60 of whom had male offspring and 55 had female offspring. The remaining 119 milk samples (33%) came from 59 mothers of twins, 23 pairs of male twins, 20 pairs of female twins, and 16 pairs of twins of different sexes.

The mean (SD) gestational age was 30.4 (3.3). Thirteen (7.5%) were mothers of infants who were small-for-gestational age.

Overall, 360 samples of milk were obtained: 241 (67%) from mothers of singletons and 119 (33%) from mothers of twins. Only the 90 samples from mothers of either only male or only female twin pairs were included in the analysis.

The number of milk samples diminished progressively over the six weeks of postpartum follow-up. The highest decrease occurred from the third week postpartum onwards. The most common reasons were direct breastfeeding, milk fortification was no longer needed, or discharge from the neonatal unit. In other cases, mothers began producing less milk, which was replaced by DHM.

In the milk of mothers of singletons, the following differences between sexes were observed:

− During the 3rd week postpartum, the milk for females had a higher mean (SD) fat and total energy content than the milk for males: 4.7 (1.2) vs. 3.5 (1.1) g/dL (*p* = 0.005), and 81.9 (12.4) vs. 67.4 (13.7) kcal/dL (*p* = 0.004), respectively (Table 1).
− During the 4th week postpartum, the milk for females had a higher mean (SD) fat and true protein content than the milk for males: 4.9 (2.1) vs. 3.6 (0.6) g/dL (*p* = 0.026), and 1.4 (0.3) vs. 1.0 (0.3) kcal/dL (*p* = 0.038), respectively (Table 1).
− During the 6th week postpartum, the milk for males had a higher mean (SD) true protein content than the milk for females: 1.3 (0.2) vs. 1.0 (0.2) g/dL (*p* = 0.005) (Table 1).

**Table 1:** Milk compositions examined from a total of 241 milk samples for either male or female preterm singletons, during the first six weeks postpartum. Bold denotes statistically significant differences.

Postpartum Week	Nutrient	Milk Samples for Males and Females	Milk Content (g/100 mL) for Males, Mean (SD)	Milk Content (g/100 mL) for Females, Mean (SD)	*p*-Value
1st week	Total protein (g)	35/38	2.21 (0.36)	2.46 (1.03)	0.173
	True protein (g)	35/38	1.75 (0.30)	1.96 (0.82)	0.153
	Fat (g)	35/38	2.63 (1.28)	2.86 (1.73)	0.520
	CHO (g)	35/38	7.55 (0.76)	7.24 (1.02)	0.153
	Energy (kcal)	35/38	64.1 (12.73)	67.2 (17.3)	0.402
2nd week	Total protein (g)	39/33	1.75 (0.52)	1.90 (0.43)	0.180
	True protein (g)	39/33	1.40 (0.41)	1.51 (0.35)	0.220
	Fat (g)	39/33	3.69 (1.53)	3.90 (1.34)	0.388
	COH (g)	39/33	6.89 (1.42)	7.35 (0.87)	0.095
	Energy (kcal)	39/33	66.9 (15.6)	71.6 (10.6)	0.150
3rd week	Total protein (g)	18/14	1.68 (0.59)	1.79 (0.41)	0.561
	True protein (g)	18/14	1.31 (0.35)	1.44 (0.32)	0.294
	Fat (g)	18/14	3.46 (1.06)	4.69 (1.22)	**0.005**
	COH (g)	18/14	7.08 (1.36)	7.66 (0.35)	0.100
	Energy (kcal)	18/14	67.4 (13.7)	81.9 (12.4)	**0.004**
4th week	Total protein (g)	9/18	1.43 (0.38)	1.71 (0.53)	0.175
	True protein (g)	9/18	1.03 (0.30)	1.37 (0.41)	**0.038**
	Fat (g)	9/18	3.59 (0.56)	4.85 (2.09)	**0.026**
	COH (g)	9/18	7.26 (1.12)	7.79 (0.95)	0.097
	Energy (kcal)	9/18	67.4 (6.89)	83.4 (22.8)	0.052
5th week	Total protein (g)	8/9	1.55 (0.56)	1.27 (0.52)	0.299
	True protein (g)	8/9	1.25 (0.42)	1.02 (0.42)	0.286
	Fat (g)	8/9	4.21 (1.62)	3.11 (1.74)	0.198
	COH (g)	8/9	7.73 (0.63)	6.60 (2.17)	0.085
	Energy (kcal)	8/9	76.6 (16.49)	61.3 (22.3)	0.133
6th week	Total protein (g)	10/10	1.57 (0.26)	2.14 (2.97)	0.554
	True protein (g)	10/10	1.25 (0.19)	0.98 (0.18)	**0.005**
	Fat (g)	10/10	3.73 (0.61)	3.98 (1.17)	0.561
	COH (g)	10/10	7.62 (0.83)	7.58 (0.49)	0.897
	Energy (kcal)	10/10	71.8 (6.6)	72.6 (12.1)	0.857

CHO—carbohydrates.

The following significant differences were observed between the milk for preterm twins and for preterm singletons:

During the 1st week postpartum:

− A higher mean (SD) fat and total energy content was found in the milk for male twins than in the milk for male singletons: 4.0 (1.7) vs. 2.6 (1.3) g/dL (*p* = 0.005) (Table 4), and 77.0 (17.7) vs. 64.1 (1.7) kcal/dL (*p* = 0.011) (Table 6), respectively.

During the 2nd week postpartum:

− A higher mean total protein, true protein, carbohydrate, and total energy content was found in the milk for male twins than in the milk for male singletons: 2.2 (0.4) vs. 1.8 (0.5) g/dL (*p* = 0.002) (Table 2), 1.8 (0.3) vs. 1.4 (0.4) g/dL (*p* = 0.002) (Table 3); 7.7 (0.8) vs. 6.9 (1.4) g/dL (*p* = 0.009) (Table 5); and 81.3 (19.5) vs. 67.0 kcal/dL (15.6) (*p* = 0.007) (Table 6), respectively.
− A higher mean (SD) fat and total energy content was found in the milk for female twins than in the milk for female singletons: 4.6 (1.9) vs. 3.7 (1.0) g/dL (*p* = 0.005) (Table 4), and 81.7 (19.7) vs. 71.6 (10.6) (*p* = 0.040) (Table 6), respectively.

During the 4th week postpartum:

− A higher mean (SD) true protein content was found in the milk for male twins than in the milk for male singletons: 1.4 (0.4) vs. 1.0 (0.3) g/dL (*p* = 0.024) (Table 3).

**Table 2:** The total protein content in the milk for preterm singletons and the milk for preterm twins, according to sex, during the first six weeks postpartum. Bold denotes statistically significant differences.

	Milk for Males					Milk for Females				
	Singletons		Twins			Singletons		Twins		
Postpartum Week	Number of Samples	Total Protein (g/100 mL) Mean (SD)	Number of Samples	Total Protein (g/100 mL) Mean (SD)	*p*-Value	Number of Samples	Total Protein (g/100 mL) Mean (SD)	Number of Samples	Total Protein (g/100 mL) Mean (SD)	*p*-Value
1st week	35	2.21 (0.36)	11	2.22 (0.49)	0.961	38	2.46 (1.03)	13	2.57 (1.37)	0.743
2nd week	39	1.75 (0.52)	15	2.21 (0.35)	**0.002**	33	1.90 (0.43)	10	1.92 (0.56)	0.906
3rd week	18	1.68 (0.60)	7	1.94 (0.32)	0.290	14	1.79 (0.41)	5	1.92 (0.48)	0.574
4th week	9	1.43 (0.38)	8	1.78 (0.46)	0.114	18	1.71 (0.53)	6	1.48 (0.25)	0.326
5th week	8	1.55 (0.56)	3	1.47 (0.15)	0.811	9	1.27 (0.52)	8	1.22 (0.48)	0.867
6th week	10	1.57 (0.26)	2	1.35 (0.07)	0.275	10	2.14 (2.98)	2	1.65 (0.35)	0.827

**Table 3:** The true protein content in the milk for preterm singletons and the milk for preterm twins, according to sex, during the first six weeks postpartum. Bold denotes statistically significant differences.

	Milk for Males					Milk for Females				
	Singletons		Twins			Singletons		Twins		
Postpartum Week	Number of Samples	True Protein (g/100 mL) Mean (SD)	Number of Samples	True Protein (g/100 mL) Mean (SD)	*p*-Value	Number of Samples	True Protein (g/100 mL) Mean (SD)	Number of Samples	True Protein (g/100 mL) Mean (SD)	*p*-Value
1st week	35	1.75 (0.30)	11	1.77 (0.39)	0.828	38	1.96 (0.82)	13	2.06 (1.10)	0.728
2nd week	39	1.40 (0.41)	15	1.77 (0.30)	**0.002**	33	1.51 (0.35)	10	1.55 (0.46)	0.781
3rd week	18	1.31 (0.36)	7	1.56 (0.26)	0.104	14	1.44 (0.32)	5	1.52 (0.37)	0.633
4th week	9	1.03 (0.30)	8	1.44 (0.36)	**0.024**	18	1.37 (0.41)	6	1.22 (0.22)	0.389
5th week	8	1.25 (0.42)	3	1.20 (0.10)	0.849	9	1.02 (0.42)	8	0.98 (0.39)	0.815
6th week	10	1.2 (0.20)	2	1.10 (0.00)	0.322	10	0.98 (0.18)	2	1.30 (0.28)	0.059

**Table 4:** The fat content in the milk for preterm singletons and the milk for preterm twins, according to sex, during the first six weeks postpartum. Bold denotes statistically significant differences.

	Milk for Males					Milk for Females				
	Singletons		Twins			Singletons		Twins		
Postpartum Week	Number of Samples	Fat Content (g/100 mL) Mean (SD)	Number of Samples	Fat Content (g/100 mL) Mean (SD)	*p*-Value	Number of Samples	Fat Content (g/100 mL) Mean (SD)	Number of Samples	Fat Content (g/100 mL) Mean (SD)	*p*-Value
1st week	35	2.63 (1.28)	11	4.03 (1.69)	**0.005**	38	2.86 (1.73)	13	3.00 (1.70)	0.802
2nd week	39	3.43 (1.30)	15	4.38(1.89)	0.090	33	3.68 (1.04)	10	4.62 (1.93)	**0.050**
3rd week	18	3.46 (1.06)	7	4.49 (1.76)	0.185	14	4.69 (1.22)	5	5.04 (2.33)	0.673
4th week	9	3.59 (0.56)	8	4.30 (1.32)	0.162	18	4.85 (2.09)	6	3.55 (0.80)	0.156
5th week	8	4.21 (1.62)	3	4.17 (0.38)	0.963	9	3.11 (1.74)	8	4.26 (0.88)	0.112
6th week	10	3.73 (0.62)	2	2.95 (0.78)	0.144	10	3.98 (1.18)	2	3.90 (0.14)	0.928

**Table 5:** Carbohydrate content in the milk for preterm singletons and the milk for preterm twins, according to sex, during the first six weeks postpartum. Bold denotes statistically significant differences.

	Milk for Males					Milk for Females				
	Singletons		Twins			Singletons		Twins		
Postpartum Week	Number of Samples	Carbohydrates (g/100 mL) Mean (SD)	Number of Samples	Carbohydrates (g/100 mL) Mean (SD)	*p*-Value	Number of Samples	Carbohydrates (g/100 mL) Mean (SD)	Number of Samples	Carbohydrates (g/100 mL) Mean (SD)	*p*-Value
1st week	35	7.55 (0.759)	11	7.51 (1.46)	0.906	38	7.24 (1.02)	13	7.11 (1.07)	0.688
2nd week	39	6.88 (1.41)	15	7.73 (0.80)	**0.009**	33	7.35 (0.87)	10	7.62 (0.99)	0.412
3rd week	18	7.08 (1.37)	7	7.49 (0.64)	0.467	14	7.66 (0.35)	5	7.90 (0.82)	0.380
4th week	9	7.02 (1.26)	8	7.53 (0.97)	0.374	18	7.77 (0.95)	6	7.85 (1.04)	0.857
5th week	8	7.73 (0.63)	3	6.93 (1.55)	0.236	9	6.60 (2.17)	8	7.61 (0.92)	0.241
6th week	10	7.62 (0.83)	2	7.95 (0.64)	0.613	10	7.58 (0.49)	2	8.10 (0.14)	0.182

**Table 6:** The total energy content in the milk for preterm singletons and the milk for preterm twins, according to sex, during the first six weeks postpartum. Bold denotes statistically significant differences.

	Milk for Males					Milk for Females				
	Singletons		Twins			Singletons		Twins		
Postpartum Week	Number of Samples	Total Energy (g/100 mL) Mean (SD)	Number of Samples	Total Energy (g/100 mL) Mean (SD)	*p*-Value	Number of Samples	Total Energy (g/100 mL) Mean (SD)	Number of Samples	Total Energy (g/100 mL) Mean (SD)	*p*-Value
1st week	35	64.14 (12.73)	11	77.0 (17.50)	**0.011**	38	67.16 (17.28)	13	67.31 (16.26)	0.978
2nd week	39	66.95 (15.58)	15	81.26 (19.47)	**0.007**	33	71.61 (10.61)	10	81.7 (19.73)	**0.040**
3rd week	18	67.39 (13.70)	7	80.14 (15.58)	0.056	14	81.93 (12.40)	5	86.80 (25.05)	0.573
4th week	9	67.44 (6.89)	8	77.63 (16.27)	0.106	18	83.39 (22.78)	6	70.83 (6.46)	0.202
5th week	8	76.63 (16.49)	3	73.00 (4.36)	0.724	9	61.33 (22.32)	8	75.13 (8.04)	0.113
6th week	10	71.80 (6.60)	2	66.50 (3.54)	0.307	10	72.60 (2.12)	2	76.00 (1.41)	0.711

## 5. Discussion

This cohort study examined differences in the macronutrient and energy content in the milk from mothers of preterm infants, during the first six weeks postpartum, according to the sex of the offspring. Significant differences were found in the milk for singleton infants of either sex. The longitudinal nature of this study, as lactation progressed, took into account the reported changes in the macronutrient and energy content of preterm milk [19]. Significant differences were found, within the same sex and between the milk for twins and the milk for singletons.

### 5.1. Differences in the Milk for Male and Female Preterm Singletons

Several studies have examined sexual dimorphism in full-term breast milk composition [8,9,10,11]. In contrast, very few studies have assessed sexual dimorphism in preterm breast milk [12,13].

It is important to acknowledge the different macronutrient and energy content of term and preterm milk. While term milk supports the growth of full-term infants, preterm milk is supposed to have a particular composition that supports the catch-up growth of preterm infants.

We found a significantly higher total energy, fat, and protein content in the mature milk (during the third and fourth weeks postpartum) for female preterm singletons, than the mature milk for male preterm singletons. However, as lactation advanced (during the sixth week postpartum), the milk for males was richer in protein than the milk for females.

Unlike these results, other authors have reported a higher total energy and fat content in the milk for males than in the milk for females [12,13].

These results closely align with the reported differences in body composition between male and female infants born prematurely, which could be in part explained by the sexual dimorphism in the MOM [22,27]. Physiological differences between sexes are evident from fetal life [24]. Recently, reference charts were created for the body composition of preterm fetuses [28]. These charts show that, during the final stages of pregnancy, males have a significantly higher percentage of fat-free mass and lower adiposity, as indicated by a lower percentage of fat mass, than females. Some characteristics of this dimorphism persist after birth. For example, preterm females tend to have greater adiposity than preterm males, who have a higher percentage of fat-free mass and faster growth rates [29,30]. One could speculate that preterm milk provides sex-specific growth advantages because it is physiologically adapted to the sex-specific postnatal changes in body composition, unlike infant formula [22,27]. However, there is still a lack of evidence as to whether preterm female infants need additional fat and energy to build higher fat reserves than males and whether preterm male infants need more protein and energy to promote greater muscle growth than females [22]. If these assumptions are confirmed, further research is needed to determine the postnatal weeks in which growth spurts or greater changes in body compartments require greater support in terms of macronutrients and energy. For example, in our study, during the fourth week postpartum, the milk of the mothers of females was richer in protein than the milk of the mothers of males, but the opposite was true at the sixth week. Whether this difference is real or due to chance or bias needs to be confirmed by larger cohort studies.

### 5.2. Differences Between the Milk for Twins and the Milk for Singletons Within the Same Sex

In males, we observed that the transitional milk (during the first week postpartum) for twins was richer in fat and energy than the milk for singletons. When the milk became mature, it became richer in energy and carbohydrates (during the second week postpartum) and in protein (during the second and sixth weeks postpartum) than the milk for singletons. In females, the mature milk for twins was richer in fat and total energy than the milk for singletons, but only during the fourth week postpartum.

The composition of milk has rarely been analyzed in the context of twin births [20]. The few studies conducted on preterm births produced inconsistent results, likely due to different methodological approaches [20]. In a pool of milk samples for preterm multiples, Congiu et al. [31] reported higher protein content and lower fat content than in milk for preterm singletons. In very preterm multiples, Borrás-Novell et al. [20] found that the milk for multiples had lower protein, fat, and energy contents than the milk for singletons. The authors acknowledge their lack of information regarding the amount of milk produced, which is expected to increase with the feeding of more than one infant. Failing to control for this factor may have introduced a bias, as higher milk production is associated with lower concentrations of macronutrients [32]. Correia et al. [21] found that the milk for twins contained less energy content than the milk for singletons. The authors explain their result by the fact that frequent breastfeeding may correlate with lower breast milk energy content, since more frequent milk extraction is usually necessary for preterm twins who are unable to suckle properly [33].

### 5.3. Strengths and Limitations

Compared with other studies evaluating sex differences in preterm breast milk [12,13,20], our study included the largest sample of both mothers and milk. Furthermore, to the best of our knowledge, this is the first study to examine the differences in the composition of preterm breast milk for twins and singletons within each sex.

Some limitations of the study need to be acknowledged. The main limitation of this study was the progressive loss of milk samples to follow-up over the six weeks postpartum, which may have introduced an uncontrolled bias. However, the attrition rate seems to be essentially random and not dependent on the study design or the research team performance. The most common reasons for loss to follow-up were direct breastfeeding, no need for fortified milk, or discharge due to favorable clinical progress. In other cases of decreased lactation or availability, MOM was replaced by DHM. Some mothers occasionally failed to bring their milk for their infants who were still in the hospital and, therefore, for analysis. This is why, for example, there were more samples in the sixth week than in the fifth. Noteworthy, it has been referred that, for a random loss of less than 60%, there might be no significant bias [34].

Due to attrition, the sample size decreased and there were not enough valid repeated measures within the timeframe of the study to perform the multivariate generalized linear model for repeated measures, which was considered the ideal statistical analysis methodology in the initial protocol, to simultaneously cover the effects of time and potential confounding factors. Therefore, it was not possible to explore the interaction between sex, gestational age, adequacy-for-gestational age, and twinning. Larger cohort studies are needed to unravel it.

Other factors that may affect breast milk composition, such as maternal socioeconomic status, maternal age, maternal nutritional status, dietary intake during pregnancy, parity, pregnancy-related morbidities, circadian rhythmicity, and galactagogue use [35], were not included in the design of this analysis based on existing, prospective, and systematically collected data, due to concerns about their accurate recording for the specific purpose of this research, which was not forecasted in the initial design of the HMB database.

Additional factors that could have biased our results include the uncontrolled heterogeneity of gestational ages included in the sample (23 to 36 weeks) and the lack of information about how much milk each mother collected at each collection time. In preterm births, it has been reported that the milk for singletons with intrauterine growth restriction may have a higher protein content and lower fat content than milk for singletons with normal growth [20,21]. Additionally, a lower concentration of macronutrients has been associated with higher milk yields [20].

### 5.4. The Practical Application of This Study’s Findings

The standard fortification is the most prevalent method used in clinical practice [5,36]. It assumes the practical but unreal assumption that breast milk has a constant protein and energy content, which carries an inherent risk of energy-protein malnutrition [5]. Two alternative individualized fortification methods, consisting of adding modular macronutrient supplements to standard fortified breast milk, are commonly used instead of standard fortification [5]. In adjustable fortification, protein intake is adjusted to the infant’s metabolic response, using blood urea nitrogen as a surrogate for protein adequacy [5]. In target fortification, the content of macronutrients in breast milk is measured to determine whether to add modular macronutrient supplements to achieve desired nutritional targets [6,7].

Most neonatal units do not have access to analyzers of human milk composition; therefore, human milk fortification is led by estimation based on data from the literature on the composition of the milk of mothers of preterm infants [37]. This is the case when using standard fortification and adjusted fortification. In these settings, being aware of how the sex of the infant and twinning can affect the breast milk’s macronutrient and energy content may help with more adequate individualization of fortification.

## 6. Conclusions

This cohort study identified differences in the macronutrient and energy content of milk from mothers of preterm infants related to the infants’ sex during the first six weeks postpartum. This assessment considered whether single or twin newborns were delivered.

In the milk for singletons, we found that the mature milk for preterm females was richer in total energy, fat, and protein than the milk for males. As lactation advanced, the milk for preterm males became richer in protein than the milk for females.

We also found that the transitional milk for preterm male twins was richer in fat and energy than the milk for preterm male singletons. When the milk became mature, it became richer in energy, carbohydrates, and protein than the milk for preterm singletons. In females, the mature milk for preterm twins was richer in fat and total energy than the milk for preterm singletons during the fourth week postpartum.

When fortifying preterm milk, it may be helpful to take into account the infant’s sex and whether she or he is a twin, or whether the singleton can affect the breast milk’s composition. This information can help individualize the fortification method.

## Data Availability

The data presented in this study may be available on request from the corresponding author. According to the institutional policy, the data are not publicly available, complying with the confidentiality for the protection of personal data.
